# Supplementary material for: A Critical View of the Application of the APEX Software (Aqueous Photochemistry of Environmentally-Occurring Xenobiotics) to Predict Photoreaction Kinetics in Surface Freshwaters
Source: Molecules. 2019 Dec 18;25(1):9. doi: 10.3390/molecules25010009 (PMC7017383; doi:10.3390/molecules25010009)
Supplement: Supplementary file 1 [file molecules-25-00009-s001.zip › 3.2.4_gcc-4.4.0.zip!/3.2.4_gcc-4.4.0/3.2.4_gcc-4.4.0/doc/octave/html/interpreter/Figure-Properties.html]

Figure Properties - Untitled


Next: Axes Properties,
Previous: Root Figure Properties,
Up: Graphics Object Properties


---

##### 15.2.2.2 Figure Properties

`nextplot`: May be one of `"new"` `"add"` `"replace"` `"replacechildren"` `closerequestfcn`: Handle of function to call when a figure is closed. `currentaxes`: Index to graphics object of current axes. `colormap`: An N-by-3 matrix containing the color map for the current axes. `visible`: Either `"on"` or `"off"` to toggle display of the figure. `paperorientation`: Indicates the orientation for printing. Either `"landscape"` or `"portrait"`.
